# Human Apolipoprotein A-I Is Associated with Dengue Virus and Enhances Virus Infection through SR-BI

**DOI:** 10.1371/journal.pone.0070390

**Published:** 2013-07-24

**Authors:** Yujia Li, Cherie Kakinami, Qi Li, Baojun Yang, Hongwei Li

**Affiliations:** Department of Microbiology, University of Hawaii at Manoa, Honolulu, Hawaii, United States of America; University of Kansas Medical Center, United States of America

## Abstract

Diseases caused by dengue virus (DV) infection vary in severity, with symptoms ranging from mild fever to life threatening dengue hemorrhage fever (DHF) and dengue shock syndrome (DSS). Clinical studies have shown that significant decrease in the level of lipoproteins is correlated with severe illness in DHF/DSS patients. Available evidence also indicates that lipoproteins including high-density lipoprotein (HDL) and low-density lipoprotein (LDL) are able to facilitate cell entry of HCV or other flaviviruses via corresponding lipoprotein receptors. In this study, we found that pre-incubation of DV with human serum leads to an enhanced DV infectivity in various types of cells. Such enhancement could be due to interactions between serum components and DV particles. Through co-immunoprecipitation we revealed that apolipoprotein A-I (ApoA-I), the major protein component in HDL, is associated with DV particles and is able to promote DV infection. Based on that observation, we further found that siRNA knockdown of the scavenger receptor class B type I (SR-BI), the cell receptor of ApoA-I, abolished the activity of ApoA-I in enhancement of DV infection. This suggests that ApoA-I bridges DV particles and cell receptor SR-BI and facilitates entry of DV into cells. FACS analysis of cell surface dengue antigen after virus absorption further confirmed that ApoA-I enhances DV infection via promoting initial attachment of the virus to cells. These findings illustrate a novel entry route of DV into cells, which may provide insights into the functional importance of lipoproteins in dengue pathogenesis.

## Introduction

Dengue virus (DV), a mosquito-borne positive sense RNA virus of the *Flaviviridae* family, is the major cause of dengue in tropical and sub tropical regions [1]. There are four distinct serotypes of DV (DV-1 to 4) that infect 50 to 100 million people annually. Disease symptoms are ranged from mild fever to severe forms of illness, dengue hemorrhagic fever (DHS) and dengue shock syndrome (DSS), which account for tens of thousands deaths primarily in school-age children [1], [2]. Currently, there is no licensed drug or vaccine available for treatment and prevention [3].

High-density lipoprotein (HDL) is known as the major groups of lipoproteins that play a central role in the efflux of excess cholesterol from peripheral tissues and transport to the liver for excretion [4]. HDL together with its protein and lipid components also possesses physiological activities that inhibit oxidation and inflammation, promote endothelial cell function, and modulate immune function [5], [6], [7]. Recently, reports have shown that HDL is able to modulate virus infectivity. In particular, HDL and low-density lipoprotein (LDL) have been found to be associated with Hepatitis C virus (HCV), a member of *Flaviviridae* family [8], [9], [10], [11]. Interaction between HDL and HCV promotes virus infectivity by facilitating virus entry through the scavenger receptor class B type I (SR-BI), the cell receptor for HDL and other lipoproteins [8], [12]. The relationship between virus infectivity and lipoprotein has also been explored in clinical studies involving DV, which have revealed a direct correlation between severe dengue disease and the change of lipoprotein profiles in plasma. Significant decrease in level of HDL was observed in severe DHF/DSS patients compared to patients with milder disease, which cloud be a potential clinical predictor for DHF/DSS [13]. However, it has not been investigated if HDL has a similar effect on DV infection as found in the case of HCV infection. In this study, we found that DV exhibits increased infectivity in presence of human serum. Such an enhancement is mediated by direct association of DV with serum apolipoprotein A-I (ApoA-I), the major protein component in HDL, which facilitates cell entry of DV via cell receptor SR-BI.

## Materials and Methods

### Cell Cultures

African green monkey kidney cells (Vero), AD-293 cells (a derivative of human embryonic kidney cell line HEK293, *Stratagene*), and two human hepatoma cell lines Huh-7 and HepG2 were grown in Dulbecco’s Modified Eagle Medium (DMEM) (*Invitrogen*) supplemented with 10% fetal bovine serum (FBS) (*Sigma Aldrich*), 100 U/ml penicillin and 100 µg/ml streptomycin (*Sigma Aldrich*). Human monocytic cell line U937 was grown in RPMI-1640 medium (RPMI-1640M) (*Invitrogen*) supplemented with 10% FBS, 100 U/ml penicillin and 100 µg/ml streptomycin. Peripheral blood mononuclear cells (PBMCs) were isolated from a healthy donor and prepared using Ficoll-Paque PLUS (*GE Healthcare*) solution. Differential migration of cells after centrifugation allowed for the separation of PBMCs from blood. Cells were then subject to several 1× Phosphate-Buffered Saline (1× PBS) wash steps, resuspended in serum-free RPMI-1640M. PBMCs were isolated by adherence and unattached cells were removed. The remaining PBMCs were cultured at 1.5×10^6^ cells/ml in 24-well polystyrene tissue culture plates using RPMI 1640M supplemented with 20% FBS, 10% human pooled serum (HS) (*EMD Millipore,* Cat# 2930149/Lot#9491K), 100 U/ml penicillin and 100 µg/ml streptomycin. All cells mentioned above were incubated at 37°C with 5% CO_2_.

### Plasmid Construction

Human ApoA-I was amplified from cDNA by PCR using forward primer 5′-ACCGATATCATGAAAGCTGCGGTGCTGACC-3′ and reverse primer 5′- GGTTCTAGACTGGGTGTTGAGCTTCTTAGT-3′. PCR products were digested with EcoRV and XbaI and cloned into p3×FLAG-CMV-14 (pFLAG, *Sigma*) to generate pApoA-I/FLAG.

### Virus Stocks

Vero cells were used to propagate DV-2 New Guinea C strain (DV). Three different DV stocks were prepared as follows: (1) Vero cells were incubated with DV for 1 hour at 37°C. After adsorption, cells were washed with 1× PBS, followed by incubation in serum-free DMEM. At 7 days post infection (dpi), cell culture medium was collected and clarified by centrifugation at 500 g for 5 min. This generates virus stock DV/SFM. (2) Human serum (HS) was added into DV/SFM stock to a final concentration of 10% to produce a virus stock referred to as DV/HSM. (3) Culture supernatant harvested from pApoA-I/FLAG-transfected AD293 cells that were grown in serum-free DMEM was added to DV/SFM in the volume as indicated, generating virus stock DV/ApoAI-FLAG.

Titers of DV/SFM stocks were determined by plaque assay as described previously [7]. Serum-free DMEM was used to adjust the volume of virus stocks to make the titers of DV/SFM, DV/HSM and DV/ApoAI-FLAG at the same level.

### Sucrose Cushion Ultracentrifugation

Virus stocks were purified by ultracentrifugation through a 30% sucrose (dissolved in 1×PBS) cushion at 25,000×rpm, 4°C for 2 h (Beckman SW28 rotor). Viral pellets were resuspended in serum-free DMEM.

### Co-immunoprecipitation Assay

AD293 cells were transfected with pApoA-I/FLAG or pFLAG using Lipofectamine^®^ 2000 (*Invitrogen*) as recommended by the manufacturer. At 4 hours post transfection (hpt), cells were washed once with 1×PBS buffer and incubated with serum-free DMEM. At 3 days post transfection (dpt), supernatants from cells transfected with pApoA-I/FLAG or cells transfected the vector only pFLAG were collected and incubated with EZview™ Red ANTI-FLAG^®^ M2 Affinity Gel (*Sigma*) at 4°C for overnight. The resulting beads were washed twice with 1×TBS buffer (50 mM Tris HCl, 150 mM NaCl, PH 7.4) and incubated with DV/SFM or DV/HSM at 4°C for over night. The co-immunoprecipitates were eluted from the beads with 1×elution buffer (0.1 M glycine HCl, pH 3.5) as recommended by the manufacturer and were analyzed by Western blotting.

### Western Blotting and Antibodies

Protein samples were separated on a 12% SDS-PAGE and transferred to a Hybond™-N nylon membrane. The membranes were blocked in wash buffer containing 1% nonfat dried milk at room temperature for overnight, then incubated with the following primary antibody (anti-ApoA-I mouse monoclonal antibody, *Abcam*; anti-DV E mouse monoclonal antibody, *Thermo Scientific*; anti-FLAG mouse monoclonal, *Sigma*; anti-Actin mouse monoclonal antibody, *Thermo Scientific*; or anti SR-BI rabbit monoclonal antibody, *Thermo Scientific*) and secondary antibody (goat anti-mouse or goat anti-rabbit horse radish peroxidase, *Thermo Scientific*) at room temperature for 2 hours. Signal was detected by using enhanced chemiluminescence substrate (*Thermo Scientific*) as recommended by the manufacturer. Relative band densities were analyzed with Image J (http://rsbweb.nih.gov/ij/download.html).

### Indirect Immunofluorescence Assay

Infected cells were washed once with 1×PBS and then resuspended in 1×PBS. Cell suspensions were added to a 10-well Teflon Printed Slide (*Electron Microscopy Sciences*), and allowed to air dry and fixed with 4% paraformaldehyde solution. DV was detected by indirect immunofluorescence assay using mouse anti-DV2 complex primary antibody (*EMD Millipore*) and FITC-conjugated goat anti-mouse secondary antibody (*EMD Millipore*). Anti-fade mounting medium (*VECTOR*) was added and DV-infected cells were visualized by fluorescence microscopy (*Olympus*).

### Dengue Virus IgG Antibody Capture ELISA

Detection of dengue virus antibodies in the human pooled serum (EMD Millipore, Cat# 2930149/Lot#9491K) was performed using Dengue virus IgG ELISA kit (Abnova) and data was analyzed as recommended by the manufacturer.

### RNA Extraction and Real-time PCR

Total RNA was extracted using TRIzol (Invitrogen) as recommended by the manufacturer. cDNAs were synthesized using a dengue specific primer [14] and oligo-dT. Quantitative real-time PCR was performed using *Eppendorf* Mastercycler ep *realplex* and KAPA SYBR FAST Universal 2× qPCR master mix (*KAPA Biosystems*). Primers that are used for real-time PCR include: DV2/capsid forward 5′- GCTGAAACGCGAGAGAAACC-3′, DV2/capsid reverse 5′-CAGTTTTAITGGTCCTCGTCCCT-3′, GADPH forward 5′-CGCTCTCT GCTCCTCCTGTT-3′, and GADPH reverse 5′-ACATCGCTCAGACACCATGG-3′. Amplifications were carried out in 20 µL reaction mixtures as described in manufacturer’s instructions. Relative DV replication was measured by analysis of real-time PCR data using the web-based service for single or multiple-gene qPCR assay (*RT^2^ Profiler PCR array analysis, SABiosciences*), in which statistical analysis was also performed to calculate p values using a Student’s t-test. Copy number of DV RNA genome in infected cells was determined by real-time PCR as described previously [14].

### SiRNA Treatment

SiRNA targeting SR-BI (siRNA SR-BI) was designed using IDT SciTools RNAi Design (http://www.idtdna.com/Scitools/Applications/RNAi/RNAi.aspx). The siRNA SR-BI (5′-GGACAAGUUCGGAUUAUUUGCUGAG-3′) was purchased from *Qiagen* and Allstars Negative siRNA (siRNA CTL) (*Qiagen*) was used as a negative control. U937 cells and Huh-7 cells were seeded in 12-well plates, and transfected with 40 pmol siRNA using Lipofectamine^®^ 2000 (*Invitrogen*) as recommended by the manufacturer. At 4 hpt, cells were washed once with 1×PBS buffer and replaced with complete medium.

### Detection of Cell Surface Dengue Antigen by FACS

Huh-7 cells were suspended in serum-free DMEM and washed once with 1×PBS buffer. Virus infection was performed by incubating Huh-7 cells with DV/SFM, DV/HSM, DV/ApoAI-FLAG at MOI of 1 respectively for 30 minutes. DV/ApoAI-FLAG was generated by incubating DV/SFM with the culture supernatant collected from AD293 cells transfected with pApoA-I/FLAG at 3 dpt. During incubation, the reaction was mixed by gently agitation every 5 min to maximize virus-cell contact. DV antigen on the cell surface was detected by indirect immunofluorescence assay using mouse anti-DV2 complex primary antibody with dilution 1∶500 (EMD Millipore) and FITC-conjugated goat anti-mouse secondary antibody with dilution 1∶1000 (EMD Millipore). Flow cytometry was performed using the FACSAria (BD Biosciences).

### Ethics Statement

The study using human blood samples was approved by the University of Hawaii Committee on Human Studies (CHS#19704), and written informed consents were obtained from the donors.

## Results

### Increased DV Infectivity is Observed in the Presence of Human Serum

**Figure 1:**
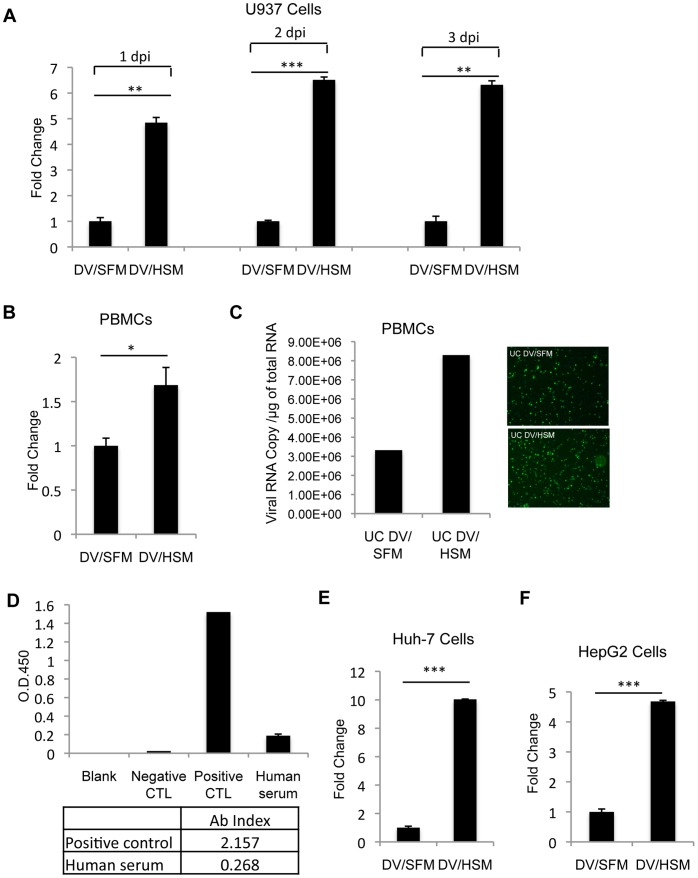
Human serum enhances infectivity of DV. (A) U937, (B) PBMCs, (E) Huh7 and (F) HepG2 cells were infected with DV collected from infected Vero cells cultured in serum-free medium (DV/SFM) or DV/SFM containing 10% human serum (DV/HSM) at an MOI of 0.1. Total RNA was extracted at 1 dpi, 2 dpi, 3 dpi for infected U937 cells respectively, and at 1 dpi for other infected cells. Viral replication was measured by real-time PCR. The results represent the average standard deviation of three independent experiments. NS, no significance; *p<0.05; **p<0.01; ***p<0.001. (C) DV/SFM and DV/HSM were ultracentrifuged (UC) over a 30% sucrose cushion, and resulting virus pellets (UC DV/SFM and UC DV/HSM) were resuspended in serum-free medium followed by infection of PBMCs. At 3 dpi, virus infection of cells was observed by IFA and virus RNA copy number was measure by real-time PCR. (D) Detection of dengue IgG antibodies in human serum. Dengue IgG ELISA kit (*Abnova*) was used to measure dengue IgG antibodies in the human pooled serum, and results were presented as antibody index (Ab index) values, which were calculated by the value of OD450 of the tested sample divided by the cut-off value that was generated from the calibrator in the kit. Ab index <0.9, no detectable IgG antibody to DV; 0.9–1.1, borderline positive; >1.1, detectable IgG antibody to DV. Positive Control (CTL) and negative control (CTL) are supplied in the kit.

In human plasma, HDL is a heterogeneous group of lipoproteins that differ in size, density and composition [15]. To determine if HDL is functionally important in the process of virus infection, we first analyzed if human serum as a whole has any effect on infectivity of DV. U937 cells were infected with DV in presence of human serum (DV/HSM) or absence of serum (DV/SFM) at a MOI of 0.1. Relative DV replication was measured by real-time PCR, and a significant enhancement of DV replication at 1, 2 and 3 dpi was observed in presence of human serum compared to absence of serum (Figure 1A). Such an enhancement was also observed in PBMCs infected with DV/HSM (Figure 1B). Because both U937 and PBMCs possess Fc receptors on the cell surface that could lead to increased virus load when presence of dengue virus antibodies at a subneutralizing level, we performed detection of dengue virus antibodies in the human serum using Dengue IgG ELISA kit (*Abnova*). Antibody index from 0.9 to1.1 is considered as the borderline positive, and Ab index of the human serum used in this study is 0.268, indicating no detectable IgG antibody to dengue virus (Figure 1D). Furthermore, we also infected Huh-7 cells and HepG2 cells with DV/SFM or DV/HSM, and virus replication was analyzed by real-time PCR at 1 dpi. Similar in U937 cells and PBMCs, presence of human serum in DV stocks leads to a significant increase of DV replication in Huh-7 and HepG2 cells (Figure 1E and 1F). As there are many proteins or factors in human serum that could potentially affect virus infection, DV/SFM and DV/HSM were further purified by ultracentrifugation over a 30% sucrose cushion, and virus pellets were resuspended in serum-free DMEM. After removal of most serum contents, the DV/HSM still exhibited infectivity that is significantly higher than DV/SFM (Figure 1C). These results suggest that certain factors present in human serum are associated with DV particles and able to promote DV infectivity.

### ApoA-I is Associated with DV

**Figure 2:**
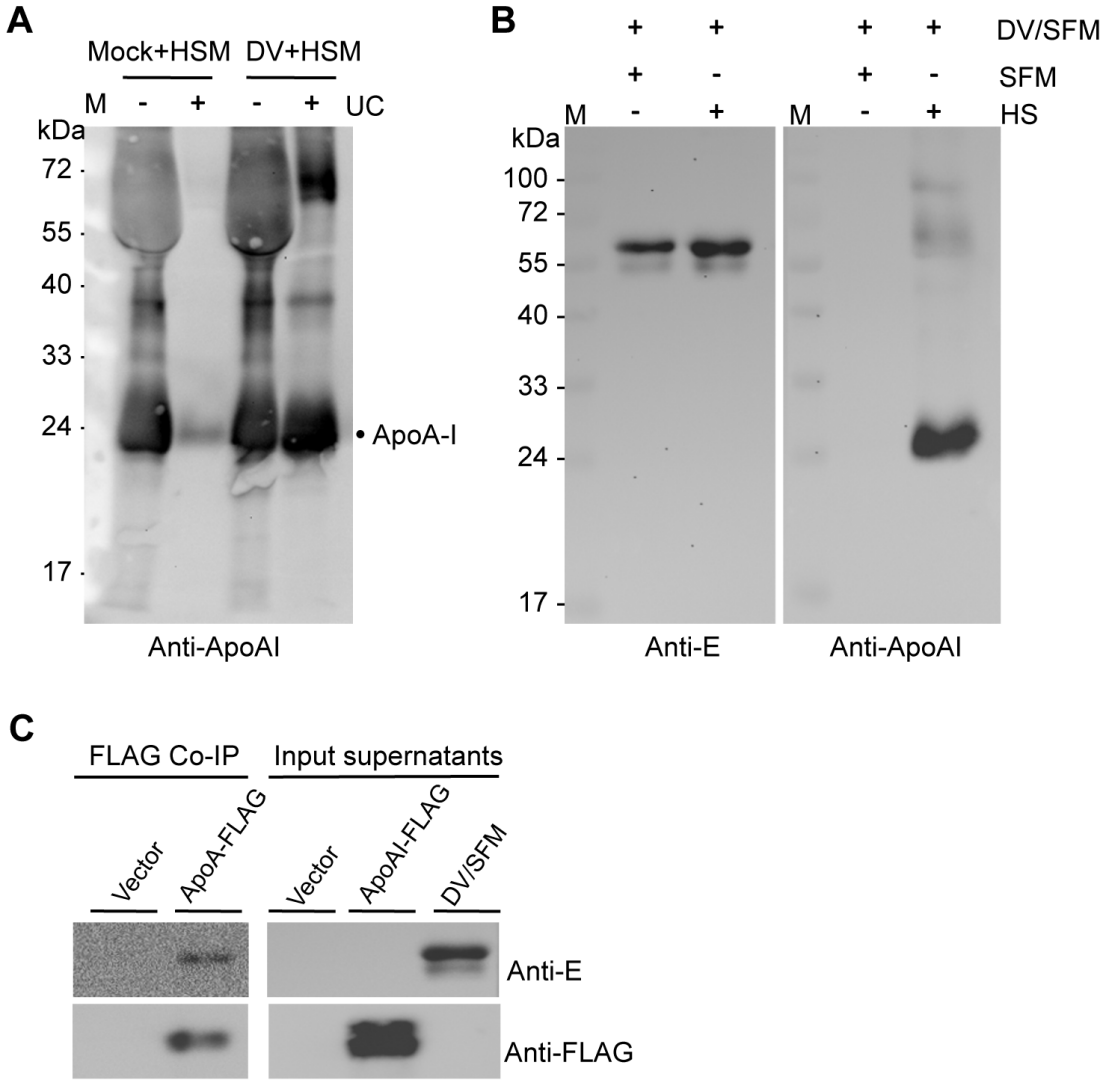
Co-precipitation of DV with ApoA-I. (A) Vero cells were infected with DV at a MOI of 1 and culture medium were changed to DMEM with 10% human serum HS (HSM) at 2 dpi. The mock-infected cells by DMEM was used as a control and also subjected to the same medium change. Culture supernatants were harvested at 7 dpi and purified by sucrose cushion ultracentrifugation (UC). The virus pellets were resuspended in serum-free DMEM. Presence of ApoA-I was analyzed by Western blotting using anti-ApoA-I antibody. (B) Human serum was added into DV/SFM to a final concentration of 10% and the mixture was incubated at 4° for 1 hour, followed by sucrose cushion ultracentrifugation. The pellets were analyzed by Western blotting using anti-ApoA-I and anti-E antibodies respectively. (C) Co-immunoprecipitation of ApoA-I with DV. AD-293 cells were transfected with a plasmid expressing FLAG-tagged ApoA-I (pApoAI-FLAG) and cultured in serum-free DMEM. At 3 dpt, secreted ApoA-I in the culture supernatant was purified with anti-FLAG M2 Affinity Gel. The resulting ApoAI-FLAG/M2 beads were washed twice with 1×TBS and incubated with DV/SFM at 4°C for over night. The co-immunoprecipitates were eluted and detected by Western blotting with anti-E and anti-FLAG antibodies. As a control, co-immunoprecipitation was also performed using the supernatant from cells transfected with empty vector p3×FLAG-CMV-14 (pFLAG). M, pre-stained protein marker.

To determine if HDL is the factor in human serum that is associated with DV and promotes virus infection, we first analyzed the presence of ApoA-I, the major protein component of HDL, in DV stock preparation. Vero cells were infected with DV and the medium was changed to DMEM with 10% human serum at 2 days post infection (dpi). As a control, Vero cells that was mock-infected was also subjected to the same medium change. Cell culture supernatants were harvested at 6 dpi and DV was concentrated by sucrose cushion ultracentrifugation, followed by resuspension of virus pellets in serum-free DMEM. Western blot analysis with anti-ApoA-I monoclonal antibody showed that only trace amount of ApoA-I was detected in the supernatant collected from mock-infected cells after ultracentrifugation. However, comparable levels of ApoA-I were detected before or after ultracentrifugation in the supernatant collected from DV-infected cells that were grown in DMEM with human serum (Figure 2A), indicating that a great amount of ApoA-I was co-precipitated with DV through ultracentrifugation. Such a correlation was also observed when DV harvested from serum-free medium (DV/SFM) was incubated with human serum (at final concentration of 10%) followed by ultracentrifugation (Figure 2B). To eliminate possible non-specific correlations between various proteins in human serum, as well as those that may arise due to the cytopathic effects generated by the virus and long incubation period, a plasmid expressing FLAG-tagged ApoA-I (pApoAI-FLAG) was constructed and transfected into AD293 cells to investigate the direct association between ApoA-I and DV. The cell culture medium was changed to serum-free DMEM at 2 hours posttransfection. At 3 dpt, significant amount of ApoA-I was secreted into the cell culture supernatant and could be detected with anti-FLAG antibody (Figure 2C). Using anti-FLAG^®^ M2 Affinity Gel, ApoAI-FLAG was purified from the culture supernatant and concentrated on anti-FLAG M2 beads. Association of DV with ApoA-I was observed by pull-down of DV from the mixture of DV/SFM with ApoAI-FLAG/M2 beads, but not in the co-immunoprecipitation of pFLAG vector (Figure 2C). This result clearly suggests a direct interaction between ApoA-I and DV.

### ApoA-I is able to Enhance DV Infectivity

**Figure 3:**
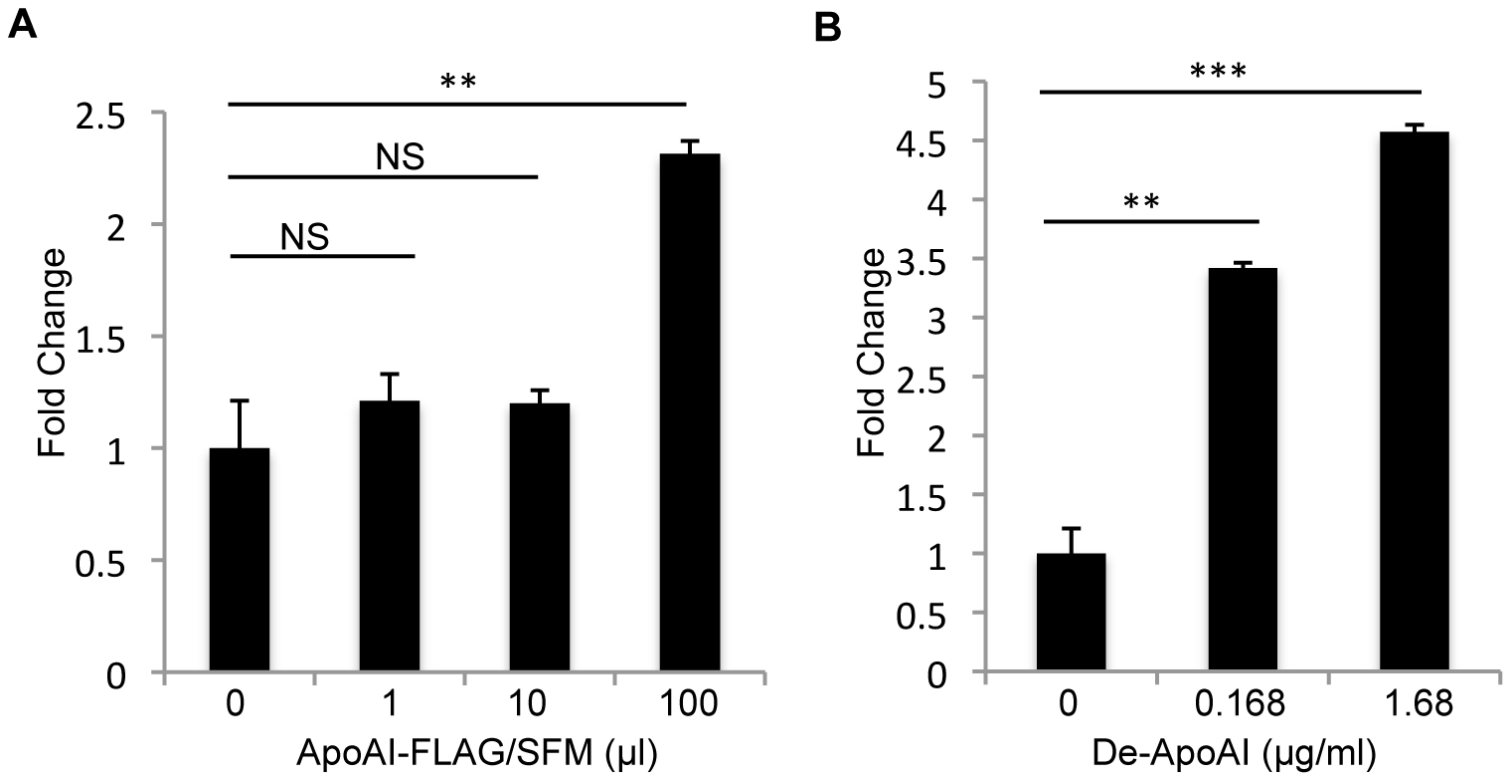
ApoA-I is able to enhance DV infectivity. DV/SFM was pre-incubated with an increasing volume of supernatants collected at 2 dpt from AD293 cells transfected with pApoAI-FLAG and grown in serum-free DMEM (ApoAI-FLAG/SFM) (A) or pre-incubated with human delipidated ApoA-I (De-ApoAI) from *Calbiochem* for 1 h at 4°C, followed by infection of U937 cells at an MOI of 0.1. Total RNA was extracted at 1 dpi, and viral replication was measured by real-time PCR. The results represent the average standard deviation of three independent experiments. NS, no significance; **p<0.01; ***p<0.001.

To determine whether ApoA-I is able to enhance DV infectivity, DV/SFM was pre-incubated with the serum-free supernatant collected from AD293 cells transfected with pApoAI-FLAG (ApoAI-FLAG/SFM). Significant increased DV infectivity was observed in U937 cells cultured in 12-well plates when presence of 100 µl of ApoAI-FLAG/SFM, but not with 1 or 10 µl, indicating ApoA-I is able to enhance DV infectivity in a dose-dependent manner (Figure 3A). In the cell culture supernatant, secreted ApoA-I is present either in a lipidated form or self-associated lipid-free form [16], [17]. To examine whether lipidation is essential for ApoA-I function in enhancement of DV infectivity, delipidated ApoA-I protein (De-ApoAI, *Calbiochem*) was used in the assay. Interestingly, 3.4 fold and 4.6 fold increase in DV infectivity was observed when DV/SFM was pre-incubated with De-ApoAI that was dissolved in serum-free DMEM at a final concentration of 0.168 µg/ml and 1.68 µg/ml (Figure 3B). Altogether, these data suggest that ApoA-I is able to enhance DV infectivity independent of lipidation.

### Complex Formation of ApoA-I and DV Prior to Infection is Important for the Enhancement of DV Infectivity

**Figure 4:**
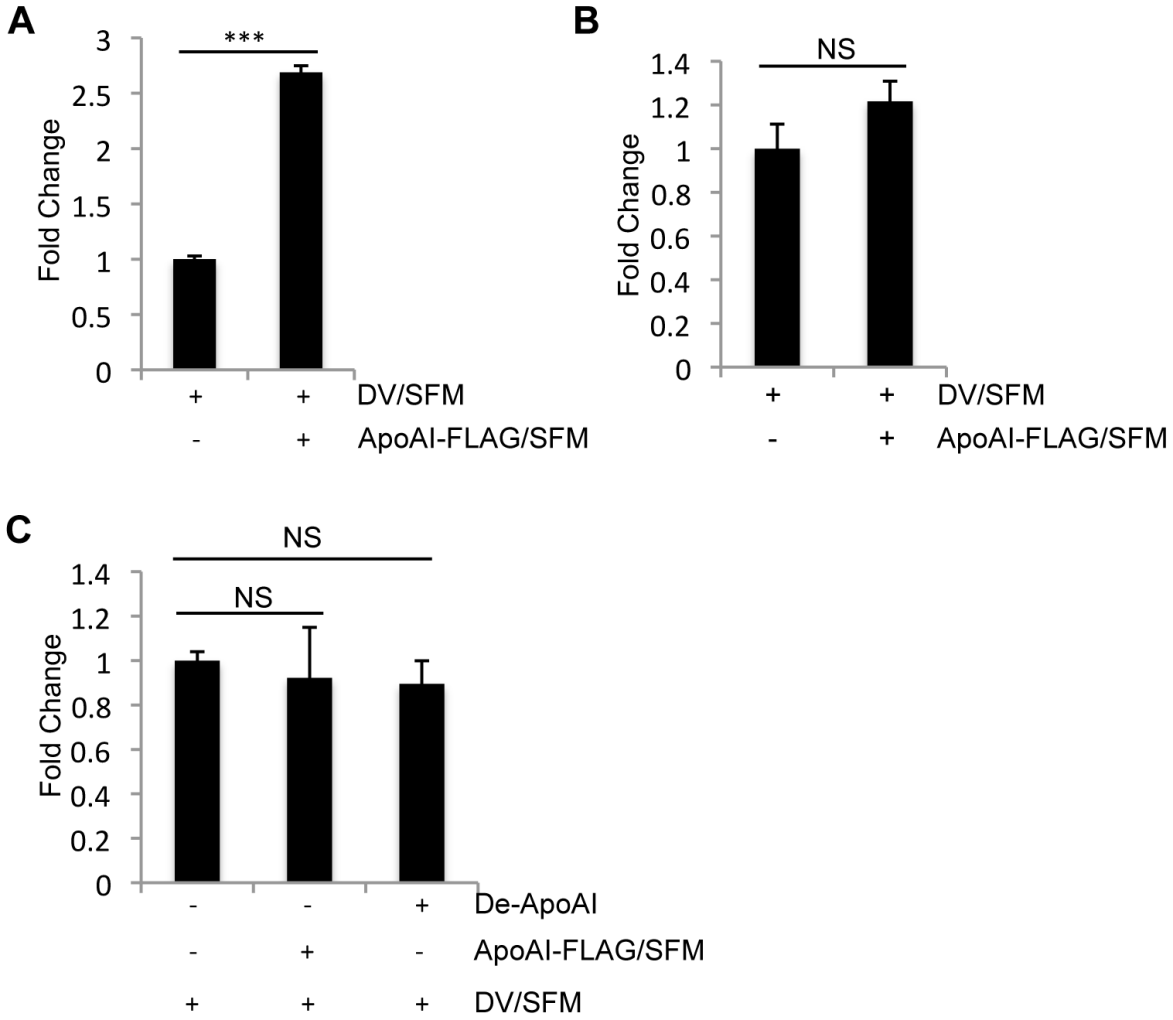
Interaction of ApoA-I and DV prior to infection is important for enhancement of virus infectivity. (A) U937 cells were infected with DV/SFM that was pre-incubated with the serum-free supernatant from ApoAI-FLAG expressing cells (ApoAI-FLAG/SFM) at 4°C for 1 hour before infection. (B) U937 cells were infected with DV/SFM in which ApoAI-FLAG/SFM was added at the time of infection. (C) U937 cells were pre-incubated with ApoAI-FLAG/SFM or De-ApoAI at 37°C for 1 h, followed by washing with 1×PBS and infection with DV/SFM. All infections were performed at an MOI of 0.1. Total RNA was extracted at 1 dpi and viral replication was measured by real-time PCR. The results represent the average standard deviation of three independent experiments. NS, no significance; ***p<0.001.

To further investigate the mode of action of ApoA-I in DV infectivity, three independent experiments were carried out. Pre-incubation of DV/SFM with 100 µl serum-free supernatant from AD293 cells transfected with pApoAI-FLAG (ApoAI/SFM) leads to significant increase of virus infectivity in U937 cells that were cultured in a 12-well plate (Figure 4A). However, no obvious effect was observed when DV/SFM was mixed with ApoAI/SFM at the time of infection (Figure 4B). Furthermore, pre-treatment of U937 cells with ApoAI/SFM or De-ApoAI (1.68 µg/ml) at 37°C for 1 h did not affect the sensitivity of U937 cells to be infected by DV/SFM (Figure 4C). Collectively, these results indicate that formation of DV/ApoA-I complex prior to infection is important for efficient enhancement of virus infection that is mediated by ApoA-I.

### Down-regulation of SR-BI Reduces DV Infection in Huh-7 Cells

**Figure 5:**
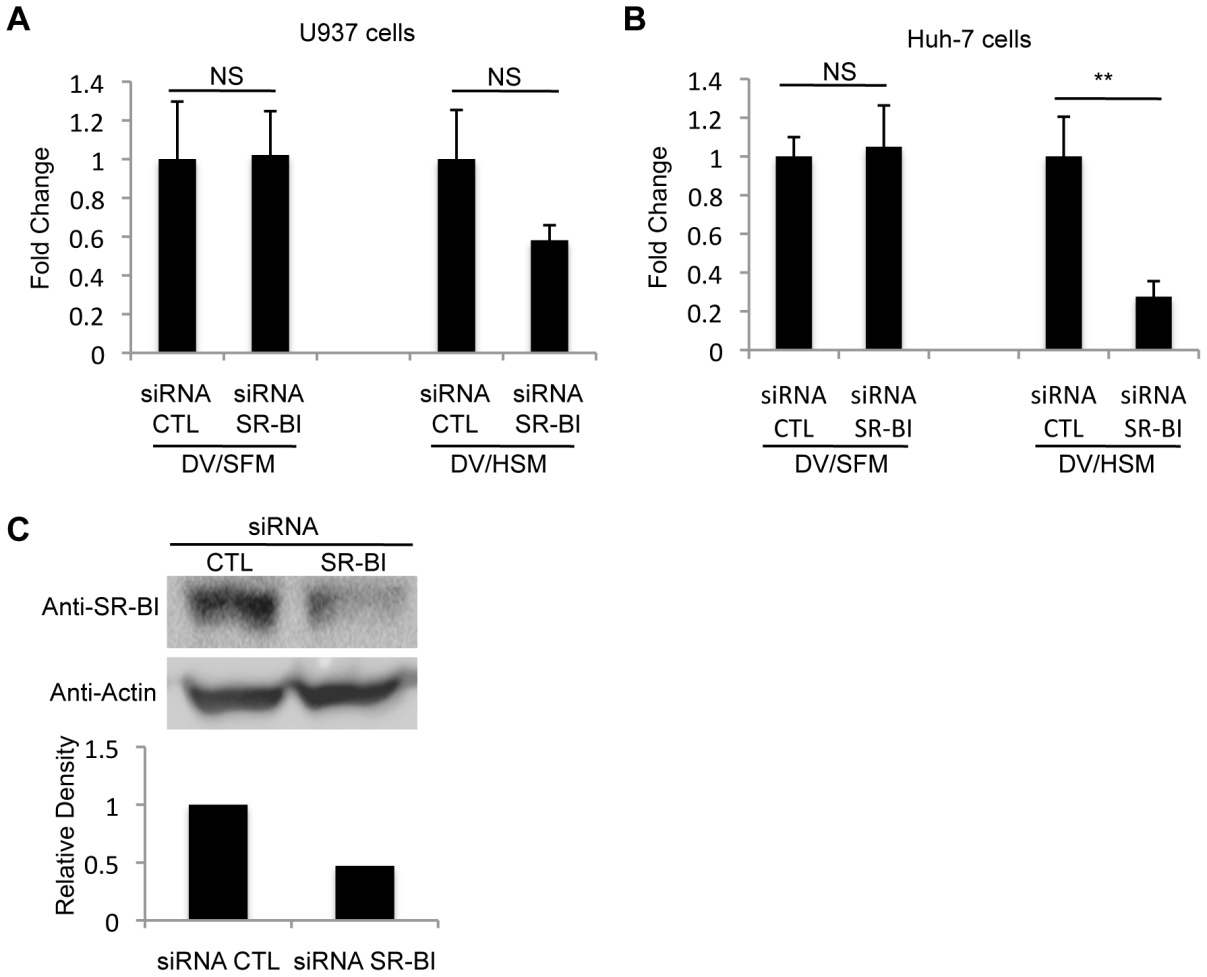
Down-regulation of SR-BI reduces DV infection in Huh-7 cells. (A) U937 cells and (B) Huh-7 cells were transfected with siRNA CTL or siRNA SR-BI. At 2 dpt, U937 cells and Huh-7 cells were infected with DV/SFM or DV/HSM at an MOI of 0.1. Total RNA was extracted at 1 dpi. Viral replication was measured by real-time PCR. The results represent the average standard deviation of three independent experiments. NS, no significance; **p<0.01. (C) Knockdown of SR-BI by siRNA. At 2 dpt, Huh-7 cell lysates were prepared and analyzed by Western blotting using anti-SR-BI and anti-Actin antibodies. The relative band density of Western blotting was analyzed with Image J.

Scavenger receptor class B type I (SR-BI) is a cell surface receptor that binds HDL and plays a central role in HDL endocytosis and cholesterol efflux [18], [19]. SR-BI is found in various types of cells or tissue, especially highly expressed in liver and adrenals [18]. It can bind protein components in different lipoproteins, such as ApoA-I in HDL and ApoB in LDL, and play an role of endocytotic receptor [20]. In the case of HCV infection, SR-BI is an essential factor for HCV entry into infected cells and such an interaction is mediated by ApoB-containing lipoproteins that are associated with HCV [8], [21], [22], [23]. Since ApoA-I is a natural ligand for SR-BI, we wondered whether the ApoA-I-mediated enhancement of DV infectivity is through SR-BI. SR-BI expression in U937 cells and Huh-7 cells was silenced using siRNA targeting SR-BI and a non-related siRNA CTL was used as a negative control. At 2 dpt, U937 cells and Huh-7 cells were infected with DV/SFM or DV/HSM at an MOI of 0.1. About 50% knockdown of SR-BI by siRNA in Huh-7 cells was confirmed by western blotting using anti-SR-BI antibody at the time of infection (Figure 5C). Analysis of virus replication by real-time PCR revealed that down-regulation of SR-BI by siRNA in Huh-7 cells significantly reduced infection of DV when presence of human serum (Figure 5B), suggesting that ApoA-I-mediated enhancement of DV infectivity is mediated by SR-BI. However, DV replication in U937 cells that were treated by siRNA SR-BI and infected with DV/HSM was decreased but not at a significant level compare to the cells treated with siRNA CTL (Figure 5A). This could be caused by relatively less expression of SR-BI in U937 cells and low transfection efficiency of U937 cells that leads to inefficient knockdown of SR-BI.

### ApoA-I Promotes DV Infection by Enhancing Virus Attachment

**Figure 6:**
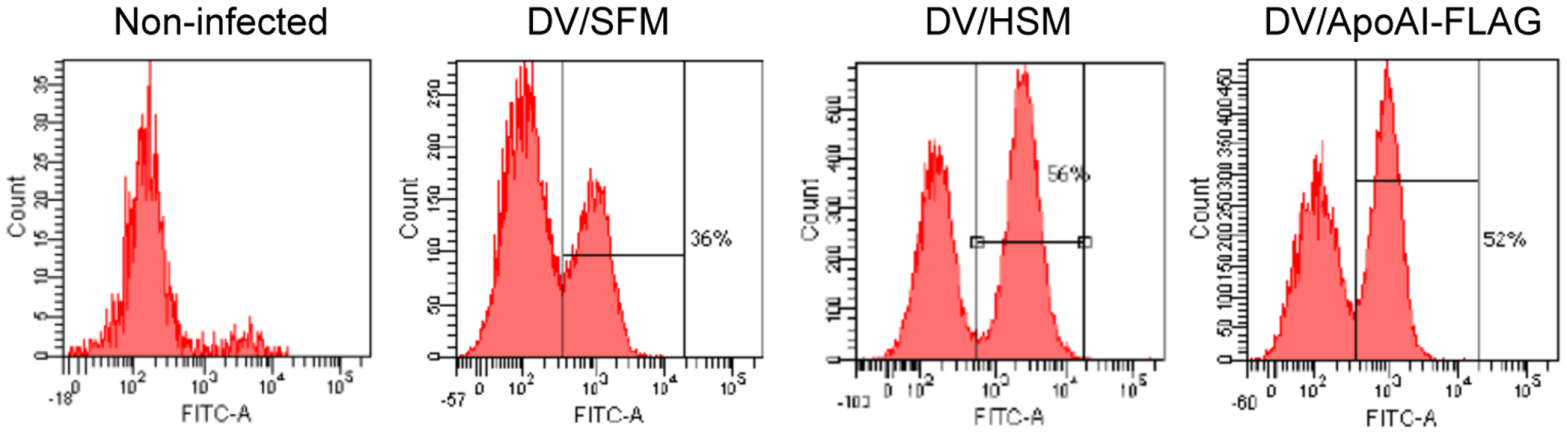
ApoA-I promotes DV attachment/entry. Huh-7 cells infected with DV/SFM, DV/HSM or DV/ApoAI-FLAG were harvested at 30 minutes postinfection. Dengue antigen on the surface of infected cells was detected by indirect immunofluorescence assay and the percentage of DV-bound cells was analyzed by FACS.

All results above indicate that ApoA-I is associated with DV, and its interaction with cell surface receptor SR-BI could bring DV to cells, which facilitates virus attachment and entry. To confirm this, detection of cell surface dengue antigen was performed by indirect immunofluorescence assay at 30 minutes after incubation of Huh-7 cells with dengue virus (DV/SFM) or dengue virus inocula containing ApoA-I, including DV/HSM and DV/ApoAI-FLAG. The immunostained cells were then analyzed by FACS. Results showed that there a significant increase in the percentage of infected cells when presence of human serum (56%) or recombinant ApoAI-FLAG (52%), compared to serum-free control (36%) (Figure 6). This clearly supports the conclusion that ApoA-I facilitates dengue virus attachment and enhances virus infection.

## Discussion

The mechanism of dengue virus entry into cells is mediated by direct fusion of the virus with the cell membrane or endocytosis [24], [25], [26], [27]. A few groups of molecules have been identified as possible cell receptor for dengue virus infection, such as carbohydrate molecules, lectins, and chaperone proteins [28], [29], [30]. The results of our study demonstrated that ApoA-I is able to increases DV infectivity and such an enhancement is mediated by a bridged interaction through association of ApoA-I with DV and interaction between ApoA-I and its cell receptor SR-B1, implicating a novel mechanism in cell entry of DV.

HDL is one of the major groups of lipoproteins present in human plasma that not only plays a central role in reverse cholesterol transport, but also exhibits a broad non-specific antiviral activity in serum, which is mainly functional through blocking virus entry [31]. However, in the case of HCV, HDL is associated with the virus particles and facilitates virus entry through the SR-BI, the major cell surface receptor of HDL that initiates or promotes HCV endocytosis [21]. Apolipoprotein E may also potentially contribute to HCV entry by promoting the initial attachment of virus to cell surface via interaction between viral envelope protein and SR-BI [32]. In our study, we found that presence of human serum leads to increased DV infection in different types of cells, in which ApoA-I was identified as a factor that influencing virus infectivity. Although it is currently not clear if ApoA-I that is associated with DV particles is present in a form of HDL, lipid-poor nascent ApoA-I or lipid free ApoA-I, our data shows that virus infectivity can be enhanced when virus was pre-incubated with secreted form of ApoA-I expressed from transfected cells or delipidated ApoA-I. This implicates possibility that nascent ApoA-I or free ApoA-I may have direct interaction with DV particles. SiRNA knockdown of SR-BI, the cell surface receptor that mediates HDL endocytosis through interaction with ApoA-I, gave rise to a compromised enhancement of DV infection in presence of ApoA-I, indicating that ApoA-I may facilitate DV infectivity via SR-BI and promote the initial attachment of virus to the cell surface.

Under conditions of infection, inflammation and various diseases, reduction of HDL and induction of serum amyloid A (SAA) in plasma are considered as the major components of acute phase response [33], [34], [35]. SAAs are mostly apolipoproteins of HDL, and able to displace ApoA-I from HDL and poses important influence in HDL structure and function during inflammation, which enhances HDL clearance [36]. Even though there is an overall decrease in ApoA-I in plasma that is accompanied by reduction of HDL [36], [37], a significant increase of lipid-free ApoA-I has been observed in the acute phase response [38]. During DV infection, a significant decrease in levels of HDL and LDL are correlated with severe cases of disease (DHF and DSS) but not with mild cases [13]. It remains unknown how the change in HDL profile may affects dengue pathogenesis. However, it is possible that increased accumulation of lipid-free ApoA-I in plasma caused by DV infection may promote complex formation of DV with lipid-free ApoA-I, which subsequently facilitates cell entry of DV and contributes to disease development. However, mechanism of interaction between ApoA-I and DV needs to be further explored.
